# Selection of peptides binding to metallic borides by screening M13 phage display libraries

**DOI:** 10.1186/1472-6750-14-12

**Published:** 2014-02-10

**Authors:** Martin Ploss, Sandra J Facey, Carina Bruhn, Limor Zemel, Kathrin Hofmann, Robert W Stark, Barbara Albert, Bernhard Hauer

**Affiliations:** 1 Institute of Technical Biochemistry, University of Stuttgart, Allmandring 31, 70569 Stuttgart, Germany; 2 Eduard-Zintl-Institute of Inorganic and Physical Chemistry, Technische Universität Darmstadt, Alarich-Weiss-Str. 12, 64287 Darmstadt, Germany; 3 Center of Smart Interfaces, Physics of Surfaces and Institute of Materials Sciences, Technische Universität Darmstadt, Alarich-Weiss-Str. 10, 64287 Darmstadt, Germany

## Abstract

**Background:**

Metal borides are a class of inorganic solids that is much less known and investigated than for example metal oxides or intermetallics. At the same time it is a highly versatile and interesting class of compounds in terms of physical and chemical properties, like semiconductivity, ferromagnetism, or catalytic activity. This makes these substances attractive for the generation of new materials. Very little is known about the interaction between organic materials and borides. To generate nanostructured and composite materials which consist of metal borides and organic modifiers it is necessary to develop new synthetic strategies. Phage peptide display libraries are commonly used to select peptides that bind specifically to metals, metal oxides, and semiconductors. Further, these binding peptides can serve as templates to control the nucleation and growth of inorganic nanoparticles. Additionally, the combination of two different binding motifs into a single bifunctional phage could be useful for the generation of new composite materials.

**Results:**

In this study, we have identified a unique set of sequences that bind to amorphous and crystalline nickel boride (Ni_3_B) nanoparticles, from a random peptide library using the phage display technique. Using this technique, strong binders were identified that are selective for nickel boride. Sequence analysis of the peptides revealed that the sequences exhibit similar, yet subtle different patterns of amino acid usage. Although a predominant binding motif was not observed, certain charged amino acids emerged as essential in specific binding to both substrates. The 7-mer peptide sequence LGFREKE, isolated on amorphous Ni_3_B emerged as the best binder for both substrates. Fluorescence microscopy and atomic force microscopy confirmed the specific binding affinity of LGFREKE expressing phage to amorphous and crystalline Ni_3_B nanoparticles.

**Conclusions:**

This study is, to our knowledge, the first to identify peptides that bind specifically to amorphous and to crystalline Ni_3_B nanoparticles. We think that the identified strong binding sequences described here could potentially serve for the utilisation of M13 phage as a viable alternative to other methods to create tailor-made boride composite materials or new catalytic surfaces by a biologically driven nano-assembly synthesis and structuring.

## Background

The diverse group of transition metal borides comprises compounds that exhibit or combine interesting physical properties such as semiconductivity, ferromagnetism, hardness, thermal and mechanical stability and oxidation resistance [1]. Some of the binary, ternary, and quaternary metal borides are even superconducting or represent some of the strongest permanent magnets [2,3]. Because of the high melting point of boron, the traditional synthesis of metal borides does not result in nanoscale or nanostructured products. As an alternative, a room temperature synthesis of nickel boride nanoparticles via hydrolysis of NaBH_4_ has been reported [4-7]. Biological systems, on the other hand, can synthesise materials under mild and environmentally benign conditions [8]. Nickel borides like Ni_3_B, Ni_2_B or NiB are considered to exhibit catalytic activity in all kinds of reduction reactions, and hydrogenation reactions [9-15]. Like iron borides they are expected to exhibit paramagnetism, ferro- or superparamagnetism [16,17]. The electrical conductivities of different nickel borides vary with their boron content. Thus, the nickel-boron system represents an interesting class of compounds for a model study on peptide-boride interactions and the potential of bio-modified inorganic solids. Until now nothing is known about the interaction between boron-containing materials and peptides, although peptides binding to solid surfaces have been extensively exploited for other chemical systems in nanoscale science because of their material selective properties [18-20]. To select for peptides that are capable of interacting with inorganic materials, phage peptide display is a powerful technique [21]. Phage display is a selection technique in which a combinatorial library of random peptides (~10^9^) is expressed as a fusion with an M13 phage coat protein (usually p3), resulting in the display of the fused peptide on the surface of the phage particle. Selection of the desired peptides, which is usually a random 7- or 12-amino acid peptide, is achieved by multiple rounds of target binding, elution and amplification, a process known as biopanning (or panning). Because the DNA sequence for the displayed peptide is genetically fused to the p3 gene, the amino acid sequence of the phage-displayed peptide is readily obtained by sequencing the p3 encoding DNA.

Phage display has been used to identify peptides which specifically bind to metals like Ag, and Pd, and various inorganic materials like ZnO, SiO_2_, TiO_2_, ZnS, and CdS [22-29]. In the last few years, specific binding peptides to nickel have also been described [30-32]. Up to now, no specific binding peptides have been identified for borides in general, neither nanostructured nor bulk material, or more specifically nickel boride nanoparticles. The identification of such a selective binding peptide sequence would be a first, significant step towards peptide-modified boride materials and composites. Such linkers would help to make boride particles available for biological systems and allow for a tuning of the properties of borides using a biological peptide-enabled nano-assembly process.

Since to our knowledge no binding peptides to boride nanoparticles are described in the literature, our aim of this research was to identify peptides that bind specifically to metallic borides. This constitutes the first step in order to combine two different binding motifs e.g. a metallic and a ceramic boride into a single bifunctional phage or into a synthetic bifunctional peptide. This would be useful for the generation of new boride composite materials in which the physical properties of two different borides are unified which is until now not combinable. Peptides were selected after several rounds of screening with a commercial phage library. Several Ni_3_B-binding peptides were selected using nanoparticles of amorphous and crystalline Ni_3_B as targets. From the initial library, we identified ten phages that exhibit selective binding to either amorphous or crystalline or both using titer count analysis, fluorescence and atomic force microscopy.

## Results and discussion

### Isolation of Ni_3_B-binding peptides

To identify Ni_3_B-binding peptides, a commercial available M13 bacteriophage display library (Ph.D.-7; New England Biolabs) was screened against amorphous and crystalline Ni_3_B nanoparticles. In the Ph.D.-7 library, random linear heptapeptides are displayed as N-terminal fusions to the minor coat protein p3 of M13. The M13 phage p3 constructs used in the screening selection have five copies of the peptide displayed on the one end of the phage particle. Five rounds of biopanning were conducted for each of the amorphous and crystalline Ni_3_B substrates. Each biopanning round involved the steps of phage binding, removal of weak binding affinity phage by washing, and elution of bound phage by a rapid pH decrease. More stringent washing conditions were used in the additional rounds of panning. A 0.3% TBST wash solution was used to remove phage with low binding affinity to Ni_3_B during the second round, whereas 0.5%, 0.6%, and 0.8% TBST wash solutions were used for the third, fourth, and fifth round, respectively. A total of 58 phage clones were selected and analysed by DNA sequencing after the fourth and fifth round of biopanning against amorphous and crystalline Ni_3_B substrates. For amorphous Ni_3_B substrates, 30 peptides were identified coding for 15 different peptide sequences (designated with an A for amorphous). A summary of the peptides identified from biopanning against amorphous Ni_3_B is provided in Table 1. In the case of the biopanning against crystalline Ni_3_B substrates, 28 peptides were isolated coding for 28 different peptide sequences (designated with a C for crystalline) shown in Table 2. The fact that all the peptides which were identified for crystalline Ni_3_B are different, could be an indication of a more complex surface structure for crystalline Ni_3_B nanoparticles than for amorphous Ni_3_B nanoparticles. Interestingly, one peptide sequence, ANHQSAN, termed A6/C28, was isolated from both, amorphous and crystalline, Ni_3_B substrates. While most of the peptides, especially identified for the crystalline Ni_3_B substrate, were isolated only once, for the amorphous substrate the peptide A1 was isolated eight-times, A2 six-times, and A3 four-times, respectively.

**Table 1:** **Summary of the isolated binding peptides to amorphous Ni**_**3**_**B**

**Peptide**	**Frequency**	**Sequence**	**Number of functional amino acid residues**			**p** ***I***
			**Basic**	**Acidic**	**Hydrophobic**	
A4	1/30	SEIVDNH	1	2	2	4.35
A1	8/30	TNLTLAS	0	0	3	5.19
A2	6/30	GALPNNL	0	0	3	5.52
A8	1/30	NVNSTSF	0	0	2	5.52
A9	1/30	SPDTVQK	1	1	1	5.55
A10	1/30	GNRLSAD	1	1	2	5.84
A7	1/30	LGFREKE	2	2	2	6.14
A11	1/30	TQVYHPM	1	0	3	6.40
A6	1/30	ANHQSAN	1	0	2	6.19
A5	1/30	TNSSFHK	2	0	1	8.44
A12	1/30	NTVIYQK	1	0	3	8.59
A13	1/30	HVQYWQF	2	0	3	8.75
A3	4/30	SLAVSRS	1	0	3	9.47
A14	1/30	VSVNSRT	1	0	2	9.72
A15	1/30	RLLNPWI	1	0	4	9.75

**Table 2:** **Summary of the isolated binding peptides to crystalline Ni**_**3**_**B**

**Peptide**	**Frequency**	**Sequence**	**Number of functionnal amino acid residues**			**p** ***I***
			**Basic**	**Acidic**	**Hydrophobic**	
C15	1/28	LEQTPMF	0	1	3	4.00
C16	1/28	ELTQISS	0	1	2	4.00
C2	1/28	SDPQTHT	1	1	0	5.06
C3	1/28	TPPLLSP	0	0	2	5.19
C17	1/28	MNHAESY	1	1	2	5.22
C18	1/28	VPSLTPT	0	0	2	5.49
C4	1/28	VPIPYLP	0	0	4	5.49
C19	1/28	DPYNRIN	1	1	2	5.84
C20	1/28	RTFDAIS	1	1	3	5.84
C21	1/28	YELVLPK	1	1	4	6.00
C5	1/28	ETFPARG	1	1	2	6.10
C13	1/28	GPVNHQL	1	0	2	6.74
C22	1/28	LNHVLPA	1	0	4	6.74
C23	1/28	HAMRTEP	2	1	2	6.75
C6	1/28	ATSTAHA	1	0	3	6.79
C28	1/28	ANHQSAN	1	0	2	6.79
C10	1/28	SYTKLI-IL	2	0	3	8.33
C1	1/28	SPPKSNA	1	0	1	8.47
C9	1/28	SASKVHN	2	0	2	8.49
C24	1/28	SPSTHWK	2	0	1	8.49
C25	1/28	WNAKYTL	1	0	4	8.59
C12	1/28	YQVVPAR	1	0	4	8.75
C26	1/28	GDPKAAR	2	1	2	8.75
C11	1/28	GDHSRHK	4	1	0	8.76
C14	1/28	AGLPKHQ	2	0	2	8.80
C27	1/28	STFNSRV	1	0	2	9.47
C8	1/28	VHTNPSR	2	0	1	9.73
C7	1/28	GASATRT	1	0	2	9.75

### Characteristics of the identified binding peptides

**Figure 1:**
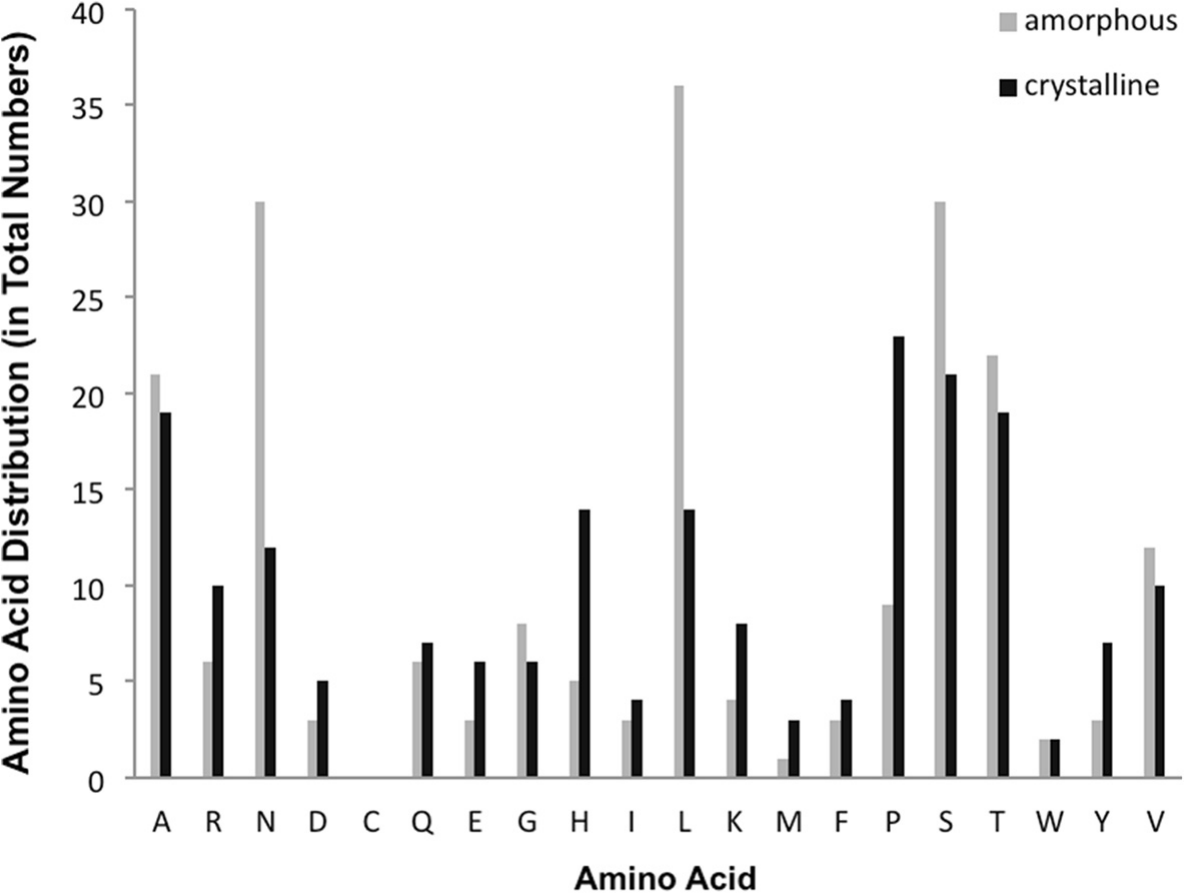
**Amino acid distribution of the isolated peptide sequences.** A total of 15 peptide sequences were analysed for amorphous Ni_3_B (grey bars) and a total of 28 peptide sequences were analysed for crystalline Ni_3_B (black bars).

Due to the fact that cysteines interfere with the p3-mediated M13 infection process, no cysteines were present in the identified binding peptides [33]. The binding peptides showed approximately three-times more positively charged residues (K, R, and H) than negatively charged residues (D and E) (Table 3). Compared with the peptides binding to amorphous Ni_3_B, the binding peptides to crystalline nickel boride show a two-times higher occurrence of the positively charged amino acids arginine and lysine, and a three-times higher occurrence of histidine, respectively. Although the distribution of charged amino acids in the library is comparable in frequency, the increased abundance of these amino acids within the identified sequences could be possibly attributed to the surface composition of the nickel borides. The characterisation of the surface of different amorphous nickel borides by Okamoto *et al.* and Caputo *et al.* by X-ray photoelectron spectroscopy (XPS) and X-ray diffraction (XRD), respectively, revealed that the electron densities on nickel are increased by electron transfer from boron to the metal [6,34]. Based on the shift of the charge, specific binding peptides could therefore interact with the nickel boride substrates via electrostatic interactions. The majority of the peptides identified on Ni_3_B prevalently possess alanine, leucine, proline, serine and threonine residues (Figure 1) which is a consequence of the amino acid distribution of the applied phage display library. In addition, the binding peptides identified on amorphous Ni_3_B possess 2.5-times more asparagine residues as compared with the peptides identified on crystalline Ni_3_B. This is vice versa with proline residues binding to crystalline Ni_3_B. The strong presence of asparagine in the peptide sequences binding to amorphous Ni_3_B, and asparagine and histidine in the peptide sequences binding to crystalline Ni_3_B suggests that the binding between the Ni_3_B and peptides could furthermore occur via hydrogen bonding interactions. The almost complete absence of tryptophan, phenylalanine, and methionine residues is also due to the low frequencies of these amino acids within the library. An amino acid position consensus was not evident from the 7-mer library screening of Ni_3_B nanoparticles as substrates. However, besides the predominantly hydrophobic amino acids (A and L) and hydrophilic amino acids (S and T) the variety of amino acids with charged side groups implies that these groups could be essential in specific binding to both substrates via electrostatic interactions. The Ni_3_B-binding peptides identified possess calculated theoretical isoelectric points (p*I*) ranging from strongly acidic (4.0) to highly basic (9.75). 60% of the peptides binding to amorphous Ni_3_B show a p*I* value < 7.5 while 40% of the peptides have p*I* > 7.5. 57% of the peptides binding to crystalline Ni_3_B show a p*I* value < 7.5 and 43% of the peptides have p*I* > 7.5. Therefore the identified peptides can be grouped into the categories acidic (p*I* 3.0 - 5.9; 35.7% of the peptides), neutral (p*I* 6.0 – 8.9; 52.4% of the peptides), and basic (p*I* 9.0 – 12; 11.9% of the peptides), respectively.

**Table 3:** **Occurrences of amino acids of the isolated Ni**_**3**_**B binding peptides**

**Amino acid**	**Averaged quantity of occurrences per heptapeptide**	
	**Amorphous**	**Crystalline**
A	0.70	0.68
R	0.20	0.36
N	1.00	0.43
D	0.10	0.18
C	**0.00**	**0.00**
Q	0.20	0.25
E	0.10	0.21
G	0.27	0.21
H	0.17	0.50
I	0.10	0.14
L	1.20	0.50
K	0.13	0.29
M	0.03	0.11
F	0.10	0.14
P	0.30	0.82
S	1.00	0.75
T	0.73	0.68
W	0.07	0.07
Y	0.10	0.25
V	0.40	0.36

### Relative binding affinity experiments

**Figure 2:**
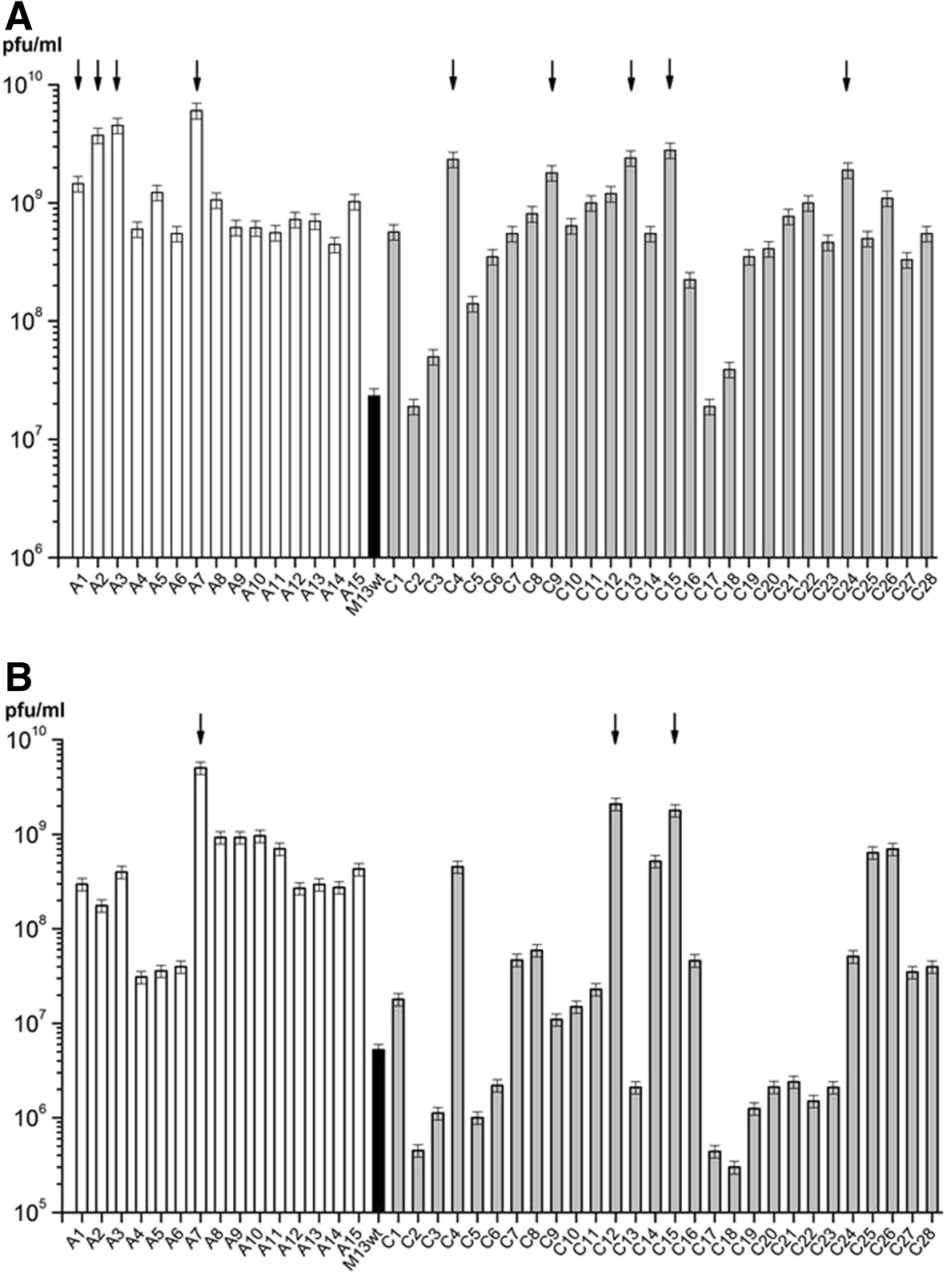
**Evaluation of the binding strength of each of the 42 identified phage clones.** The relative binding affinity of the phage clones to amorphous **(A)** and crystalline **(B)** Ni_3_B nanoparticles was determined by titer assays at pH 7. The assay was repeated three-times for each clone and the elucidated phage amounts were arithmetically averaged. As a control, M13KE wild-type (M13wt) phage without a random peptide insert were compared in the same manner as the phage clones. Strong binding phage clones (> 10^9^ pfu/ml) are indicated with an arrow. Clones identified on amorphous Ni_3_B are designated with an A, clones identified on crystalline Ni_3_B are designated with a C.

Because of the heterogeneity of all of the identified binding peptides and the absence of a distinctive binding motif, the binding strength of each of the 42 identified peptides to amorphous (Figure 2A) and crystalline (Figure 2B) Ni_3_B was determined by titer assays. Since previously binding affinity experiments at pH 5 or pH 9 with a subset of the identified binding peptides showed no increase in binding affinity (data not shown) the relative binding affinity experiments were carried out at pH 7. As a control to verify that the peptide was the interactor of interest, M13KE wild-type (M13wt) phage without a random peptide insert were compared in the same manner as the peptide clones. As presented in Figure 2A, most of the phage clones, except C2 and C17, bind to amorphous Ni_3_B with greater affinity than does the M13wt phage, which shows a binding affinity of 2 × 10^7^ pfu/ml with amorphous Ni_3_B. Compared with the M13wt phage, 9 out of 42 identified peptides (see arrows in Figure 2A) bound on average 100-times and 28 on average ten-times more efficiently to amorphous Ni_3_B than the wild-type, verifying that binding to the substrate was a result of the peptide sequence and not due to non-specific phage coat protein interactions. The peptides A7, C12, and C15 (see arrows in Figure 2B) bound on average 1000-times more efficiently to crystalline Ni_3_B as compared to the M13wt phage, which shows a binding affinity of 5 × 10^6^ pfu/ml. Interestingly, the phage clone A7 identified for amorphous Ni_3_B shows a higher binding affinity to crystalline Ni_3_B than most of the Ni_3_B-binding peptides (C1-C11, C13-C14, and C16-C28) identified for crystalline Ni_3_B. In addition, the amorphous Ni_3_B-binding peptides A8-A10 show a higher binding affinity to crystalline Ni_3_B (≥10^9^ pfu/ml) than several binding peptides found for crystalline Ni_3_B specifically (i.e. C1-C11, C13-C14, C16-C24, and C27-28). The relative binding affinity experiments revealed a set of several strong binders (>10^9^ pfu/ml) for amorphous (A1-A3, A7, C4, C9, C13, C15, and C24) and crystalline (A7, C12, and C15) substrates (Table 4). The phages displaying the peptides A7 and C15 emerged as the best binders for both substrates.

**Table 4:** Strong binders identified by relative binding affinity experiments

**Ni**_**3**_**B amorphous target**					
**#**	**Peptide**	**Frequency**	**Phage titer (pfu/ml)**	**Sequence**	**p** ***I***
1	A7	1/30	6.1 × 10^9^	L G F R E K E	6.14
2	A3	4/30	4.6 × 10^9^	S L A V S R S	9.47
3	A2	6/30	3.8 × 10^9^	G A L P N N L	5.52
4	C15	1/28	2.8 × 10^9^	L E Q T P M F	4.00
5	C13	1/28	2.4 × 10^9^	G P V N H Q L	6.74
6	C4	1/28	2.4 × 10^9^	V P I P Y L P	5.49
7	C24	1/28	1.9 × 10^9^	S P S T H W K	8.49
8	C9	1/28	1.8 × 10^9^	S A S K V H N	8.49
9	A1	8/30	1.5 × 10^9^	T N L T L A S	5.19
**Ni**_**3**_**B crystalline target**					
**#**	**Peptide**	**Frequency**	**Phage titer (pfu/ml)**	**Sequence**	**p** ***I***
1	A7	1/30	5.1 × 10^9^	L G F R E K E	6.14
2	C12	1/28	2.1 × 10^9^	Y Q V V P A R	8.75
3	C15	1/28	1.8 × 10^9^	L E Q T P M F	4.00

### Competitive binding assays of the best binding peptides

The competitive binding assay represents a method to determine the most selective binding peptide sequence from a large subset of phage display results [35]. To identify a specifically recognised structural correlation between the surfaces of amorphous and crystalline Ni_3_B nanoparticles, the individual phage clones of the nine strongest binders (A1-A3, A7, C4, C9, C13, C15, and C24) to amorphous Ni_3_B as determined by the relative binding affinity experiments were diluted into a single mini-phage library. The mini-phage library was then screened against amorphous or crystalline Ni_3_B substrates. To receive better statistical results, the competitive binding assays were repeated three-times. After a single panning experiment, the strongly bound phages were isolated and a total of 58 phage clones were randomly selected and analysed by DNA sequencing to identify the strongest binding peptides. In Tables 5 and 6 the most abundant sequence indicates the strongest binding peptide to amorphous and crystalline Ni_3_B, respectively. Among the 58 randomly chosen plaques, the phage displaying the A7 peptide LGFREKE has the strongest binding affinity to amorphous (seventeen-times, ~58%) and crystalline (thirteen-times, ~45%) Ni_3_B. Interestingly, the amino acids lysine, arginine, and glutamic acid were shown to have a pronounced adhesion of their charged side groups to amorphous Si_3_N_4_ and SiO_2_ under aqueous conditions [36]. It was also reported, that the surface of amorphous Si_3_N_4_ may present negative sites in solution [37]. Therefore substrates, possessing negatively surface charges in aqueous solution with pH values less than the intrinsic pK_a_ of the side chain of a basic amino acid, a peptide containing basic amino acids will be positively charged at these groups and will provide an attractive interaction to the given surface [36]. In aqueous solutions with higher pH values than the intrinsic pK_a_ of the side chain of an acidic amino acid, a peptide containing acidic amino acids will be negatively charged at these groups. Based on these facts, the best binder A7 (LGFREKE) possesses in solution at pH 7 an alternating series of positively and negatively charged side chains in its peptide sequence which could be responsible for an interaction between the charge depleted boron and the electrons accumulated on the nickel side of amorphous and crystalline Ni_3_B nanoparticles. In contrast, the 7-mer peptide sequence TNLTLAS, termed A1, which emerged as the dominant amorphous Ni_3_B binder in the initial screening, possesses no charged side chain. The lack of electrostatic interactions might contribute to the observed fact, that it does not show the highest frequency in the competitive assay.

**Table 5:** **Competitive binding assay results for amorphous Ni**_**3**_**B target**

**Peptide**	**Sequence**	**Frequency**
A7	L G F R E K E	17/29
C24	S P S T H W K	3/29
C9	S A S K V H N	2/29
C15	L E Q T P M F	2/29
A1	T N L T L A S	1/29
A2	G A L P N N L	1/29
C4	V P I P Y L P	1/29
C13	G P V N H Q L	1/29
A3	S L A V S R S	0/29

**Table 6:** **Competitive binding assay results for crystalline Ni**_**3**_**B target**

**Peptide**	**Sequence**	**Frequency**
A7	L G F R E K E	13/29
C9	S A S K V H N	5/29
C24	S P S T H W K	5/29
C13	G P V N H Q L	4/29
C4	V P I P Y L P	1/29
C15	L E Q T P M F	1/29
A1	T N L T L A S	0/29
A2	G A L P N N L	0/29
A3	S L A V S R S	0/29

### Fluorescence microscopy of Ni_3_B-M13 complex

**Figure 3:**
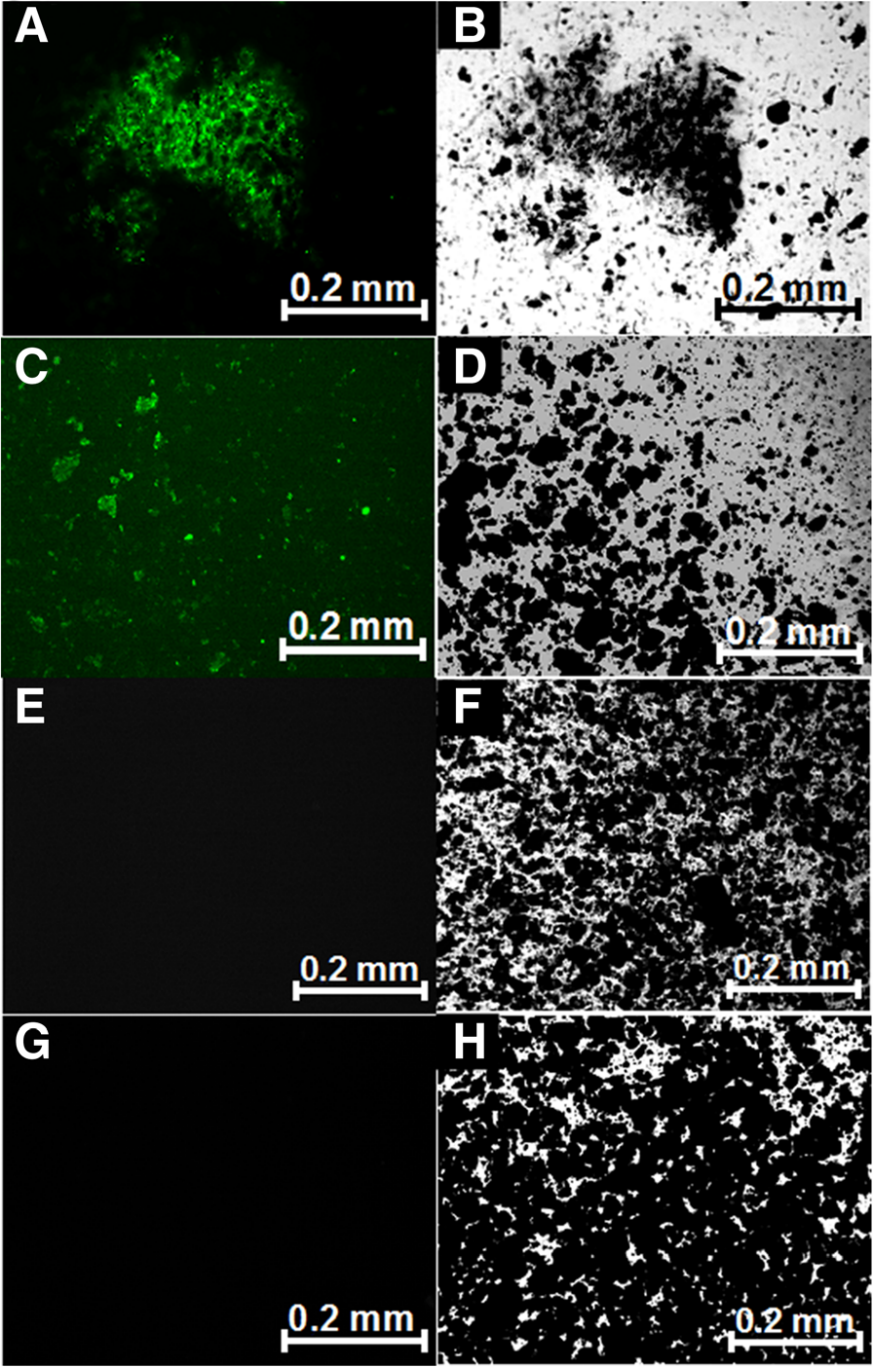
**Analysis of the phage binding by fluorescence microscopy.** Confocal fluorescence **(A, C)** and transmission optical microscopy **(B, D)** of LGFREKE phage bound to amorphous **(A, B)** and crystalline **(C, D)** Ni_3_B nanoparticles. The samples were dyed using a fluorescently tagged anti-M13 monoclonal antibody. Confocal fluorescence **(E, G)** and transmission optical microscopy **(F, H)** of the control experiments of amorphous and crystalline Ni_3_B nanoparticles, respectively, which were preincubated with M13KE wild-type (M13wt) phage before incubation with the fluorescently tagged antibody.

The binding of phage displaying the A7 peptide LGFREKE to amorphous and crystalline Ni_3_B nanoparticles was confirmed by fluorescence microscopy. Amplified phage displaying the LGFREKE peptide were incubated either with amorphous or crystalline Ni_3_B nanoparticles and then washed to remove the nonspecifically bound phage particles. The samples were then dyed using a fluorescently tagged anti-M13 monoclonal antibody and washed again to eliminate nonspecifically bound anti-M13 antibodies. The fluorescence confocal images in Figure 3A and C show the LGFREKE displaying phage binding to the surface of amorphous and crystalline Ni_3_B nanoparticles, respectively. Considerable fluorescence emits from the surfaces of the LGFREKE phage-Ni_3_B nanoparticles. Although the relative binding affinity experiments showed an unspecific binding of the wild-type phage to the Ni_3_B substrates, the control experiments (Figure 3E and G) showed no fluorescence. This could be due to the fact that the concentration of bound phage is below the detection limit.

### AFM measurements

**Figure 4:**
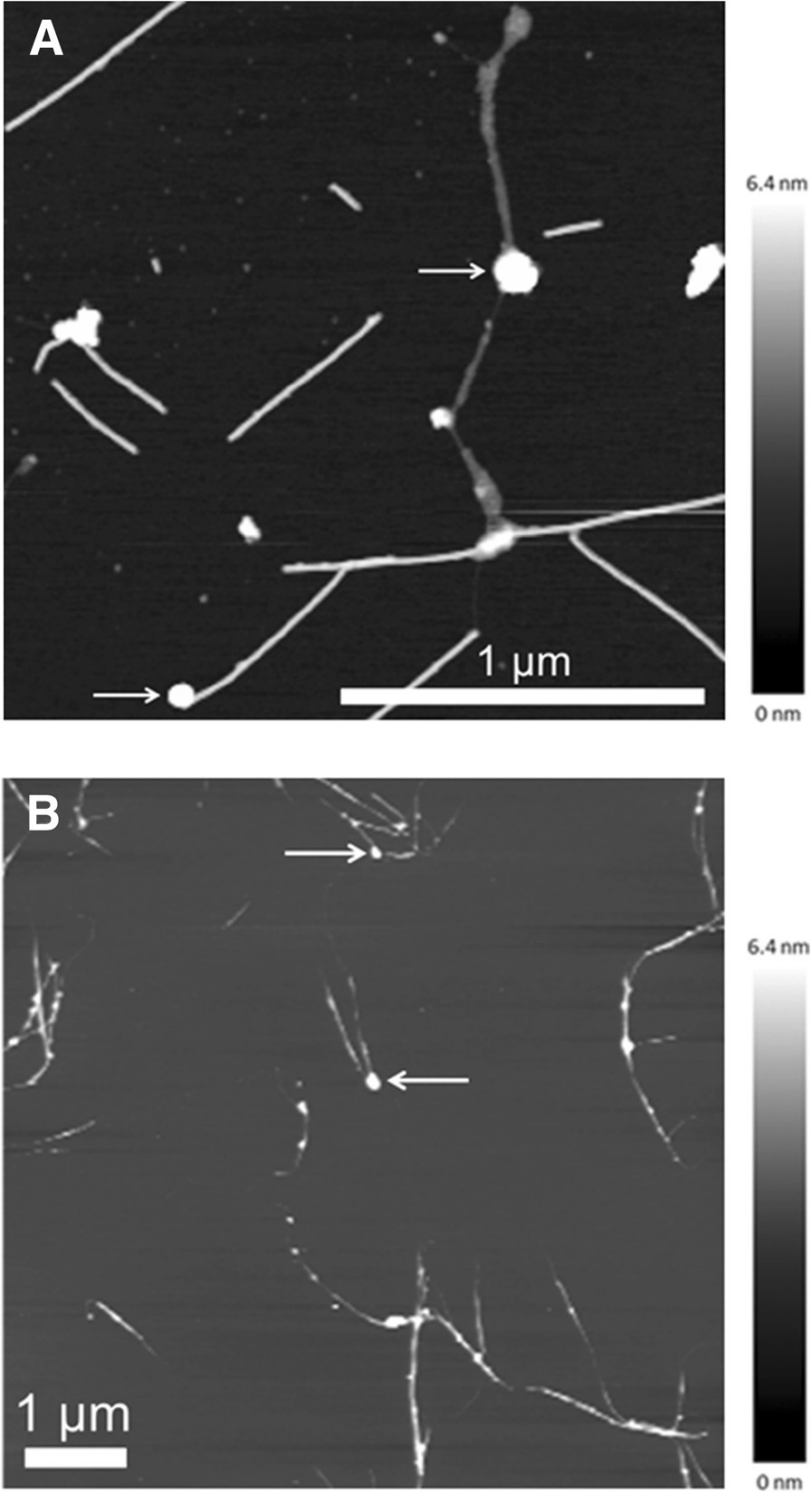
**Visualisation of the phage binding by atomic force microscopy.** Topographic AFM images of the binding of modified M13 phage (LGFREKE) to amorphous **(A)** and crystalline **(B)** Ni_3_B nanoparticles. The AFM images of M13 phage displaying the A7 peptide on p3 showed the phage to bind to several amorphous and crystalline Ni_3_B nanoparticles at the tip of p3, which are indicated by arrows. The colour scale exceeds the particle height to better display the particle cross section.

To verify the interaction between M13 phage displaying the A7 peptide on the p3 coat protein and Ni_3_B particles, AFM measurements of phage bound to amorphous and crystalline Ni_3_B particles were carried out (Figure 4). The modified M13 phage were incubated either with amorphous or crystalline nanoparticles as shown in Figure 4A and B, respectively. As shown in Figure 4A and B the height of the colour scale exceeds the height of the particles. This colour scale was chosen to show the 100-nm-thick particles together with the 6-nm-thick phage. Individual phage are approximately 1 μm in length and 6.5 nm in height. This cross section and length of the modified phage correspond to that of M13 wt phage (data not shown). Some phage were oriented with their ends towards a nanoparticle (see arrows in Figure 4), which implies an affinity between the modified phage proteins and the particle. There are a few particles that seem to be attached to the unmodified side of the phage. Possible explanations for this observation include unspecific interaction or the presence of more than one phage, i.e. bundles of phage where the end of one phage coincides with the side of another phage. A small level of unspecific interaction between nanoparticles and phage cannot be avoided during preparation for AFM imaging. The phage oriented with their ends towards the nanoparticles, suggest that the peptide LGFREKE interacts with both amorphous and crystalline Ni_3_B nanoparticles.

## Conclusions

Metal borides are inorganic solids that are very interesting due to their high versatility in physical properties, as for example magnetism, electrical and thermal conductivity, hardness and catalytic activity, but at the same time they are difficult to prepare. Besides the application as catalysts in all kinds of reduction reactions, and hydrogenation reactions, metallic borides are employed for the generation of conductive ceramics, hard magnets, superconductors, and hard materials [9-15,38-40]. Typical synthesis routes employ high reaction temperatures and do not allow for a directed synthesis of particles that are well-defined in terms of size, shape, uniformity, phase purity, or crystallinity. Formation of metal boride nanoparticles in solvents has been described occasionally, but an interaction of borides with biological material or bio-molecules like peptides was unknown before [4,5]. To the best of our knowledge, the present study is the first to identify peptides that bind specifically to amorphous and to crystalline Ni_3_B nanoparticles. Although all identified peptide sequences showed no significant binding motif, the analysis of the resulting Ni_3_B-binding sequences found them to be enriched in charged amino acids. The identified binding sequences possessed approximately three times more positively charged than negatively charged amino acids which leads to the assumption of electrostatic interactions between the identified binding peptides and the Ni_3_B substrates. Fluorescence microscopy and AFM studies directly confirmed the adhesion of the best binding phage displaying the peptide LGFREKE to amorphous and crystalline Ni_3_B particles. The best binding peptide sequence could play an important role for the development of a new synthesis route of Ni_3_B in the presence of phage displaying the peptide, or the synthetic peptide, respectively, which would represent a complete new approach. The synthesis of Ni_3_B in the presence of LGFREKE could result in mono-disperse and peptide capped nanoparticles with well-defined and uniform sizes and shapes exhibiting new catalytic and magnetic properties. Furthermore, the utilisation of M13 phage as template for the Ni_3_B mineralisation could potentially serve as a viable alternative to create tailor-made boride composite materials by a biologically driven nano-assembly synthesis and structuring for the generation of thermoelectric devices and field effect transistors.

## Methods

### Phage and bacteria strains

Recombinant M13 phage from the Ph.D.-7 heptapeptide library (New England Biolabs, MA) were amplified and propagated in the *E. coli* host strain ER2738 (F', *lacI*^*q*^, Δ(*lacZ*)*M15*, *proA*^*+*^*B*^*+*^_,_*zzf::Tn*10(Tet^R^)/*fhuA2*, *supE*, *thi-1*, Δ(*lac-proAB*), Δ(*hsdS*-*mcrB*)5, [r_k_^-^m_k_^-^*McrBC*^-^]), as described by the manufacturer. M13KE wild-type (M13wt) phage was used for the determination of unspecific binding to the Ni_3_B targets. Bacteria cultures were maintained in lysogeny broth (LB) [41].

### Peptide library screening

Ni_3_B-binding peptides were identified using a commercially available M13 bacteriophage display library (Ph.D.-7; New England Biolabs) with random 7-mer peptides. The DNA sequences of the 7-mer peptides are N-terminal fusions to the minor coat protein p3 of the bacteriophage M13. A short linker sequence (GGGS) separates each random peptide from the minor coat protein p3. The phage display library consists of approximately 1.28 × 10^9^ different peptides. The target binding, elution, and phage amplification steps were conducted according to the manufacturer’s instructions. 20 mg of amorphous or crystalline Ni_3_B nanoparticles were used as targets. The targets were washed three-times with Tris-buffered saline (TBS, 50 mM Tris–HCl, pH 7, and 150 mM NaCl) and blocked with TBS supplemented with 5 mg/ml of BSA for 1 hour. The blocked targets were then washed three-times with TBS. To each target, a total number of 2 × 10^11^ phage in 1 ml of TBS with 0.1% Tween-20 (0.1% TBST) were incubated for 1 hour. Non-specifically or weakly bound phage particles were removed by washing ten-times with 0.1% TBST. The remaining bound phage particles were eluted with a solution containing 0.2 M glycine-HCl, pH 2.2 and 1 mg/ml BSA. After centrifugation, the supernatant was removed from the target and neutralised with 1 M of Tris–HCl, pH 9. The eluted phage were amplified through infection into *E. coli* ER2738, followed by purification by precipitation with polyethylene glycol according to the manufacturer’s instructions. This completed a round of panning. The entire biopanning process was repeated five rounds to enrich for tight binders. To inhibit the hydrophobic interactions between the phage library and the Ni_3_B targets, the concentration of Tween-20 was successively increased in each panning step from 0.1%, 0.3%, 0.5%, 0.6%, and 0.8%, respectively. After the fourth and fifth round of screening, the eluted phage were diluted and titrated on LB agar plates containing 5-bromo-4-chloro-3-indolyl-β-D-galactopyranoside (Xgal) and isopropyl-β-D-thiogalactopyranosid (IPTG). A total of 58 randomly selected phage plaques, which appeared blue, were picked and analysed by DNA sequencing. The phage titer was determined by titration according to manufacturer’s recommendations using appropriate dilutions of the precipitated phage.

### Binding affinity assays

The binding affinity of each identified phage clone with the Ni_3_B substrates was demonstrated by mixing equivalent phage amounts (1 × 10^10^ pfu/ml) of individual phage clones with either 20 mg of amorphous or crystalline Ni_3_B nanoparticles in 1 ml of TBS containing 0.8% Tween-20. After 1 hour incubation at room temperature the unbound phage were removed by washing the substrates ten-times. Bound phage were then isolated using the standard acidic elution method as used in the screening procedure. For each clone, the bound phage amount was elucidated by titration according to manufacturer’s recommendations using appropriate dilutions of the eluted phage. The binding strength of a single clone directly correlates with the phage titer of the eluted phage. The assay was repeated three-times for each clone and the elucidated phage titers were arithmetically averaged. M13KE wild-type phage, with no random peptides displayed on the p3 protein, was used as a control.

### Competitive binding assays

The phages with the highest binding affinity to either amorphous or crystalline Ni_3_B were evaluated by the method of a competitive binding assay. For this assay, the individual phage clones of the nine strongest binders on amorphous Ni_3_B as determined by binding affinity assays, were separately amplified and diluted into a mini-phage library at a concentration of 1 × 10^7^ pfu/μl per clone. Subsequently 1 × 10^10^ pfu/ml of this minimised phage library was applied to amorphous or crystalline Ni_3_B nanoparticles as described for the procedure of the binding affinity assays. After a single panning round, the weakly bound phages were removed by washing the substrates and the remaining strongly bound phages were isolated using the standard acidic elution method as used in the screening procedure. The eluted phages were quantified by titration according to the manufacturer’s recommendations. The assay was repeated three-times for each substrate. A total number of 29 plaques were randomly picked for each substrate and sequenced to reveal the most strongly binding peptide sequences.

### Synthesis and characterisation of amorphous and crystalline Ni_3_B

The Ni_3_B nanoparticles were synthesised from NaBH_4_ (Alfa Aesar, 97%) and NiCl_2_ × 6 H_2_O (Grüssing GmbH, 98%) in aqueous solution at about 0°C following a synthesis route described earlier [5]. According to this procedure amorphous Ni_3_B is formed primarily, which is transformed into the crystallised Ni_3_B sample at 490°C in vacuum (5 × 10^-3^ mbar, 18 h). The X-ray diffraction pattern of the annealed, crystalline sample allows for its identification as Ni_3_B (see Additional file 1). In previous work, we have shown using methods like X-ray diffraction, energy-dispersive X-ray spectroscopy, X-ray absorption spectroscopy, scanning and transmission electron microscopy, and energy-loss electron spectroscopy that the corresponding amorphous phase actually consists of the same boride as the crystalline modification [5,7]. For the phage display the amorphous Ni_3_B nanoparticles were used after extensive washing with deionized water and drying in a desiccator under vacuum. The crystalline nanoparticles were obtained from the amorphous product after the annealing process and used directly.

### Fluorescence microscopy analysis

The binding of M13 phage to amorphous and crystalline nickel boride nanoparticles was characterised with a Zeiss Axiovert 200 inverted fluorescence microscope equipped with a Zeiss AxioCam HRm. The fluorescence images were taken using emission at 528 nm. M13 phage were incubated with 10 mg of amorphous and crystalline Ni_3_B nanoparticles at a concentration of 3 × 10^12^ pfu/ml in 1 ml TBS supplemented with 0.8% Tween-20. After 2 h at room temperature, unbound phage were removed by washing the substrates three-times with TBS. Subsequently, the substrates were blocked with TBS supplemented with 2% BSA and 0.05% Tween-20 for 1 hour and washed three-times with TBS supplemented with 0.05% Tween-20. The samples were dyed using a FITC tagged anti-M13 monoclonal antibody against phage coat protein p8 (Acris Antibodies, Inc., Herford, Germany). Each target was incubated for 1 hour with 1 μg/ml of labeled monoclonal antibodies in TBS with 0.05% Tween-20. After removing unbound antibodies by washing the samples three-times with TBS containing 0.05% Tween-20, the samples were analysed by fluorescence microscopy.

### Atomic force microscopy (AFM) analysis

Ni_3_B nanoparticles, phages and compounds of phages with Ni_3_B were characterised with an atomic force microscope (AFM). The measurements were carried out on a Dimension Icon AFM with a Nanoscope V controller (Bruker, Santa Barbara, California) in ambient conditions. The measurements were performed in tapping mode, to minimise the lateral interaction. Silicon cantilevers (PPP NCH, Nanosensors) with a nominal resonance frequency of 330 kHz, spring constant of 42 Nm^-1^, and a nominal tip diameter of less than 10 nm were used. All images were taken at a resolution of 512 × 512 pixel^2^. M13 phages were incubated with 10 mg of amorphous and crystalline Ni_3_B nanoparticles at a concentration of 3 × 10^12^ pfu/ml in 1 ml TBS supplemented with 0.8% Tween-20. After 2 h, unbound phages were removed by washing the substrates three-times with TBS. Samples were prepared by dropping the sample suspension on a silicon wafer or freshly cleaved mica. The samples were dried in a stream of dry nitrogen. Image analysis and processing was performed with the Nanoscope Analysis software Version 1.40 (Bruker). A plane correction procedure and a line by line fit were used to compensate for the sample tilt.

## Competing interests

The authors declare that they have no competing interests.

## Authors’ contributions

SJF and MP drafted the manuscript. CB and KH synthesised the Ni_3_B substrates for the use in biopanning. MP identified and characterised the binding peptides, and visualised the adhesion of the phage by fluorescence microscopy. LZ did the AFM analysis of Ni_3_B nanoparticles, phages and compounds of phages with Ni_3_B. BA, SJF, RWS, and BH planned and designed this project. SJF, MP and BA read and approved the final manuscript. All authors read and approved the final manuscript.

## Supplementary Material

Additional file 1 **X-ray powder diffraction profile.** Calculated (solid blue line) and difference (solid red line) X-ray powder diffraction profiles for the Rietveld refinement of crystalline Ni_3_B. Reflection positions are marked (blue bars). Click here for file
