# Supplementary material for: Selection of peptides binding to metallic borides by screening M13 phage display libraries
Source: BMC Biotechnol. 2014 Feb 10;14:12. doi: 10.1186/1472-6750-14-12 (PMC3924706; doi:10.1186/1472-6750-14-12)
Supplement: Additional file 1 — X-ray powder diffraction profile. Calculated (solid blue line) and difference (solid red line) X-ray powder diffraction profiles for the Rietveld refinement of crystalline Ni3B. Reflection positions are marked (blue bars). [file 1472-6750-14-12-S1.pdf]

Additional file 1

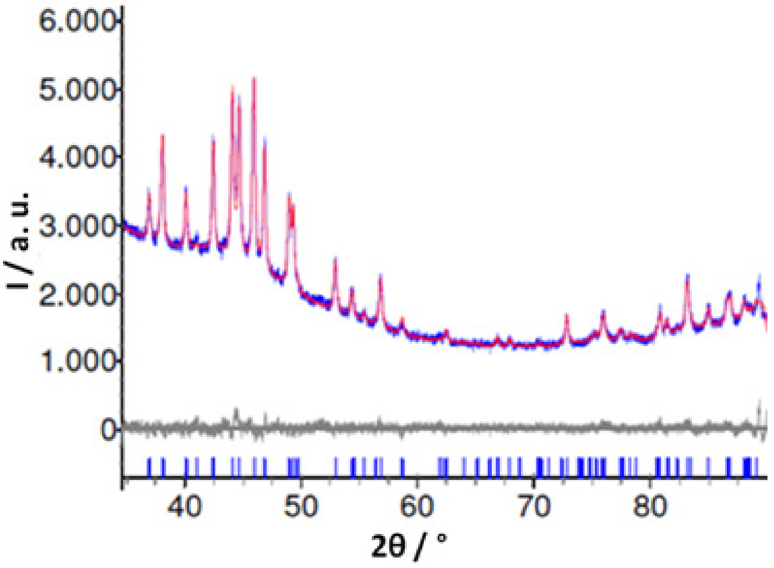

Calculated (solid blue line) and difference (solid red line) X-ray powder diffraction profiles for the Rietveld refinement of crystalline Ni_3B. Reflection positions are marked (blue bars).
